# Mechanical versus manual chest compressions in the treatment of in-hospital cardiac arrest patients in a non-shockable rhythm: a randomised controlled feasibility trial (COMPRESS-RCT)

**DOI:** 10.1186/s13049-018-0538-6

**Published:** 2018-08-30

**Authors:** Keith Couper, Tom Quinn, Ranjit Lall, Anne Devrell, Barry Orriss, Kate Seers, Joyce Yeung, Gavin D. Perkins, On behalf of the COMPRESS-RCT collaborators, Catherine Baldock, Christopher Bassford, Roger Beadle, Sukhdeep Dosanjh, Anthony Freestone, Jonathan Hulme, Catherine Lawrence

**Affiliations:** 1 Warwick Clinical Trials Unit, Warwick Medical School, University of Warwick, Coventry, CV4 7AL UK; 2 University Hospitals Birmingham NHS Foundation Trust, Birmingham, UK; 3 Emergency, Cardiovascular and Critical Care Research Group, Faculty of Health, Social Care and Education, Kingston University, London and St George’s, University of London, London, UK; 4 University of Warwick, Coventry, UK; 5 Warwick Research in Nursing, Warwick Medical School, University of Warwick, Coventry, UK

**Keywords:** Cardiac arrest, Cardiopulmonary resuscitation, Advanced cardiac life support, Mechanical chest compression device

## Abstract

**Background:**

Mechanical chest compression devices consistently deliver high-quality chest compressions. Small very low-quality studies suggest mechanical devices may be effective as an alternative to manual chest compressions in the treatment of adult in-hospital cardiac arrest patients. The aim of this feasibility trial is to assess the feasibility of conducting an effectiveness trial in this patient population.

**Methods:**

COMPRESS-RCT is a multi-centre parallel group feasibility randomised controlled trial, designed to assess the feasibility of undertaking an effectiveness to compare the effect of mechanical chest compressions with manual chest compressions on 30-day survival following in-hospital cardiac arrest.

Over approximately two years, 330 adult patients who sustain an in-hospital cardiac arrest and are in a non-shockable rhythm will be randomised in a 3:1 ratio to receive ongoing treatment with a mechanical chest compression device (LUCAS 2/3, Jolife AB/Stryker, Lund, Sweden) or continued manual chest compressions. It is intended that recruitment will occur on a 24/7 basis by the clinical cardiac arrest team. The primary study outcome is the proportion of eligible participants randomised in the study during site operational recruitment hours. Participants will be enrolled using a model of deferred consent, with consent for follow-up sought from patients or their consultee in those that survive the cardiac arrest event.

The trial will have an embedded qualitative study, in which we will conduct semi-structured interviews with hospital staff to explore facilitators and barriers to study recruitment.

**Discussion:**

The findings of COMPRESS-RCT will provide important information about the deliverability of an effectiveness trial to evaluate the effect on 30-day mortality of routine use of mechanical chest compression devices in adult in-hospital cardiac arrest patients.

**Trial registration:**

ISRCTN38139840, date of registration 9th January 2017.

**Electronic supplementary material:**

The online version of this article (10.1186/s13049-018-0538-6) contains supplementary material, which is available to authorized users.

## Background

Each year, approximately 35,000 patients have a cardiac arrest in UK hospitals, of which less than one in five patients survives to leave hospital [1]. Observational studies report the association between high-quality cardiopulmonary resuscitation (CPR) and improved survival, as well as the challenge of delivering high-quality manual CPR in clinical practice [2–5].

Mechanical chest compression devices consistently deliver high-quality chest compressions [6]. In the out-of-hospital setting, large, high-quality randomised controlled trials have found no evidence of improved patient outcome in patients treated with mechanical CPR (mech-CPR), compared with manual CPR (man-CPR) [7–10]. In contrast, very low-quality small randomised and observational studies report evidence of an association between mech-CPR and increased survival in in-hospital cardiac arrest (odds ratio of hospital/30-day survival 2.34, 95% confidence interval 1.42 to 3.85) [11].

This apparent discrepancy in findings between the in-hospital and out-of-hospital setting may reflect differences in evidence quality or clinical factors, such that mech-CPR is more effective than man-CPR in the hospital setting [12, 13]. Examples of such factors include opportunity for early optimal device deployment by a clinical team with frequent cardiac arrest exposure and the challenge of delivering effective manual chest compressions on a hospital bed, due to the compressibility of the underlying mattress [2, 14, 15].

Based on this ongoing uncertainty, there is a need for a clinical trial to evaluate the effect of the routine use of mech-CPR, compared with man-CPR, on 30-day survival in adults that sustain an in-hospital cardiac arrest. However, such a trial would require a large sample size and might be beset by a number of practical challenges, such that is prudent to first undertake a feasibility trial.

## Methods/design

**Fig. 1:**
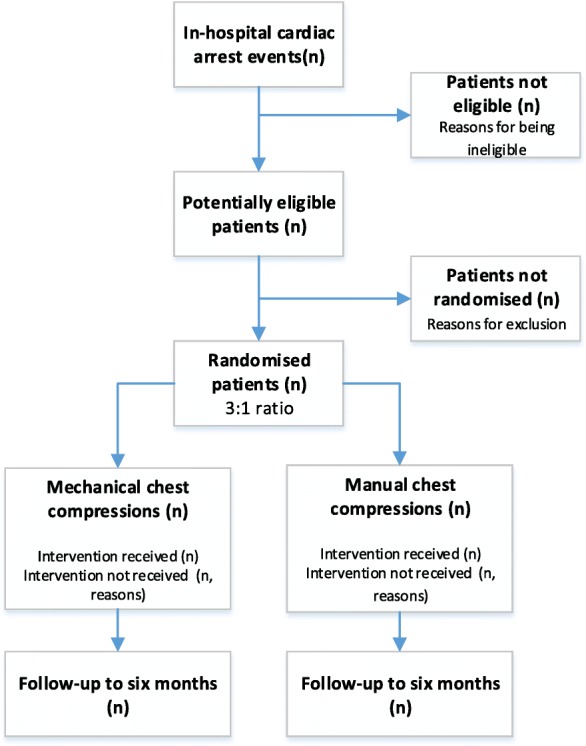
Trial flow diagram

COMPRESS-RCT is a multi-centre parallel group feasibility randomised controlled trial, in which adult patients that sustain a non-shockable in-hospital cardiac arrest are randomised in a 3:1 ratio to receive either mech-CPR or ongoing man-CPR (Fig. 1). The aim of the trial is to assess whether it is feasible to undertake an effectiveness trial to examine the effect of the use of mech-CPR on 30-day survival following in-hospital cardiac arrest. A qualitative study is embedded in the trial, in which semi-structured interviews are conducted with clinicians involved in the trial to explore potential facilitators and barriers to recruitment. The trial flow diagram is presented in figure one.

### Trial approvals and conduct

COMPRESS-RCT is approved by the West Midlands – Coventry and Warwickshire National Health Service Research Ethics Committee (16/WM/0299). The Health Research Authority Confidentiality Advisory Group has approved the processing and transfer of data without consent, under The Health Service (Control of Patient Information) Regulations 2002 (16/CAG/0088). The trial is registered with the ISRCTN Trial Registry (ISRCTN08233942). The trial is funded by a National Institute for Health Research Post-Doctoral Research Fellowship (PDF-2015-08-109).

The trial is conducted in accordance with the Medical Research Council (MRC) guidelines on Good Clinical Practice, applicable UK legislation and the Warwick University Clinical Trials Unit Standard Operating Procedures. The trial sponsor is the University of Warwick.

The study is co-ordinated by Warwick Clinical Trials Unit. It is run in collaboration with five English acute hospital research sites: Heartlands Hospital, University Hospitals Birmingham NHS Foundation Trust; University Hospital, University Hospitals Coventry and Warwickshire NHS Trust; Warwick Hospital, South Warwickshire NHS Foundation Trust; Sandwell General Hospital, Sandwell and West Birmingham Hospitals NHS Trust; and Blackpool Victoria Hospital, Blackpool Teaching Hospitals NHS Foundation Trust.

### Outcome measures

The primary outcome for this feasibility trial is the proportion of eligible patients randomised during site operational recruitment hours.

We will measure a range of secondary outcome measures, grouped as study feasibility outcomes, patient outcomes, process outcomes, and safety outcomes, which are summarised in Table 1.

**Table 1:** Study secondary outcomes

Study feasibility outcomes (formulae detailed summary in Additional file 1)	
• Proportion of patients randomised outside of weekday daytime hours). • Device deployment time- measured as the pause in chest compressions associated with device deployment. • Proportion of patients/consultees providing agreement to ongoing study participation. • Success of study blinding procedures. • Proportion of patients with complete follow-up data. • Proportion of patients with analysable chest compression quality data.	
Patient outcomes	
• Return of spontaneous circulation- defined as the return of a spontaneous circulation for at least twenty minutes. • Duration of critical care and hospital stay. • Survival- measured at hospital discharge, 30-days, and 6-months. • Quality of life- measured using EQ-5D-5 L (EuroQOL- 5 dimensions- 5 levels) and SF-12 (12-item short form survey) questionnaires at hospital discharge and 6-months. • Good neurological outcome measured using cerebral performance category (CPC) at discharge and the modified Rankin score (mRS) at discharge and at six-months. Good neurological outcome will be defined as a CPC of 1 or 2 or return to baseline (pre-admission) CPC, or mRS of 0–3 or return to baseline (pre-admission) mRS.	
Process outcomes	
• Cardiopulmonary resuscitation (CPR) quality (chest compression rate, chest compression depth, flow-fraction, pre-shock pause, post-shock pause, peri-shock pause).	
Safety outcomes	
• Device related adverse events	

### Eligibility criteria

Participants are eligible to be included in the trial if they:

1. Sustain an in-hospital cardiac arrest and resuscitation is attempted by a hospital cardiac arrest team trained in the use of the mechanical chest compression device (excludes cardiac arrests in the emergency department).
2. In a non-shockable rhythm (pulseless electrical activity or asystole) at the time of eligibility assessment.
3. Known or believed to be aged 18 years or over.

A team is categorised as being trained in device use if at least two clinicians present have been trained in device use.

Exclusion criteria are:

1. Patient has valid do not attempt cardiopulmonary resuscitation order.
2. Known or clinically apparent pregnancy.
3. Cardiac arrest where use of a mechanical chest compression device is contra-indicated (e.g. following cardiac surgery, thoracic trauma, patient size).
4. Known previous study participation.
5. Patient requiring use of mechanical chest compression device as part of routine clinical care.
6. Patient known to be detained by Her Majesty’s Prison Service.

### Recruitment and randomisation

It is intended that, where possible, the recruitment process is led 24-h a day by the hospital cardiac arrest team. On arrival of the mechanical device at the cardiac arrest location, a trained member of the cardiac arrest team assesses patient eligibility for study inclusion. If a patient is identified as being eligible, then the patient proceeds to randomisation.

Patients are individually randomised in a 3:1 ratio in favour of the use of the mech-CPR, using a sequentially numbered sealed opaque envelope system. An unequal randomisation ratio was chosen to increase clinician exposure to device use during the trial. A single envelope is stored with each mechanical device at all times. The randomisation sequence was created by the study statistician, using the centre as strata and using random blocks to ensure that a 3:1 allocation was maintained for both interventions for a given strata. Envelopes are packed by a Warwick Clinical Trials Unit staff member who is independent of the study team.

At the point that the envelope is opened, the patient is categorised as being randomised for the intention-to-treat analysis. Following envelope use, the next sequentially numbered envelope is allocated to that device.

### Consent

Cardiac arrest is a sudden, unpredictable event, in which the patient immediately loses consciousness. Furthermore, the need for immediate treatment makes it impractical to obtain informed consent from the patient’s consultee prior to trial enrolment. On this basis, a Research Ethics Committee has approved the initial enrolment of trial participants under a waiver of consent in accordance with the English law.

Participants that survive the cardiac arrest are approached at the earliest reasonable opportunity following the cardiac arrest event. Following explanation of the trial, the participant or their consultee is offered one of three options: 1) use of routine health data sources for data collection and completion of questionnaires at discharge and six-months; 2) use of routine health data sources for data collection without questionnaire completion; or 3) declination of further study involvement and data collection. In the event that the participant lacks capacity to make decisions, this approach is made to the participant’s designated personal or professional consultee.

We consulted in detail with a group of patient research ambassadors regarding the approach that should be adopted in relation to trial information provision to family members in the event that the participant dies. The group carefully considered the three main options: active information (e.g. letter); passive information (e.g. poster in hospital bereavement office), or no information. The patient research ambassadors recommended that family members should not be informed about trial participation. In forming this view, the group considered factors such as the nature of the intervention being studied and the balance of benefit and harm in providing information at a time of bereavement. This reflects the approach that has been adopted in similar trials [8, 16, 17].

### Sources of bias

#### Compliance and contamination

Compliance with allocated study treatment is being monitored, together with reasons for non-compliance, throughout the trial. It is acknowledged that non-compliance may be unavoidable in some circumstances, such as where a participant has return of spontaneous circulation following randomisation and prior to initiation of mech-CPR. Where possible, defibrillator download data is used to corroborate compliance information recorded on the case report form.

Potential causes of contamination are dependent on study allocation. In the mech-CPR arm, contamination is most likely attributable to compliance issues. In the man-CPR arm, crossover may occur if a special circumstance arises following randomisation where local policy requires use of mech-CPR (e.g. post-randomisation decision for intra-arrest coronary angiography). Such events are likely to be rare and are recorded and monitored throughout the trial.

### Blinding

The nature of the intervention makes it impossible to blind clinical personnel present at the cardiac arrest event. It is not possible to blind clinical and site research teams to treatment allocation as the intervention is recorded in the participant’s medical record and clinical examination may indicate device use (e.g. chest bruising). However, it is not considered likely that knowledge of treatment allocation will influence delivery of other intra-arrest or post-arrest interventions.

Participants will be initially blinded as they will be unconscious throughout the cardiac arrest event. An active attempt is made to maintain this blinding, although participants may nevertheless subsequently become unblinded (e.g. if they access their medical records). The rationale for maintaining blinding will be explained during the consent process and in study information. The success of participant blinding is measured through study questionnaires distributed at hospital discharge and six-months by asking participants whether they are aware of their study allocation and which treatment they believe that they received.

### Trial interventions/treatment

All patients initially receive manual chest compressions. Following commencement of the trial intervention, the intervention will continue for the duration of the cardiac arrest event. Aside from the trial intervention being tested (method of chest compression delivery), it is expected that all cardiac arrest interventions in both groups is delivered in accordance with Resuscitation Council (UK) guidelines [18].

### Mech-CPR (intervention group)

In participants randomised to the mechanical chest compression trial arm, a LUCAS-2 or LUCAS-3 device (Jolife AB/Stryker, Lund, Sweden) is deployed as early as possible following randomisation. The device is used in place of manual chest compressions as long as continued resuscitation is indicated.

Cardiac arrest teams are trained to minimise pauses in chest compression delivery during device deployment. The device is deployed in two phases (1- insertion of the back plate; 2- deployment of the upper part of the device), with chest compressions delivered between the two phases. Teams aim for the maximum chest compression pause during each phase to be less than ten seconds. The training method being used is modelled on our preparatory manikin work [19].

### Man-CPR (control group)

In patients randomised to the man-CPR arm, patients continue to receive manual chest compressions. Where available, the cardiac arrest team may use a real-time audiovisual feedback device to guide the quality of manual chest compressions.

### Data collection and management

Study data are collected by site research teams. Anonymised data are transferred securely to the trial co-ordinating centre. Study data items and definitions match, as far as possible, those used for the UK-based National Cardiac Arrest Audit [1]. Table 2 shows an overview of data to be collected and the corresponding assessment time-point. In participants that consent to follow-up, the period of follow-up is six months after the cardiac arrest event.

**Table 2:** Schedule of intervention delivery and data collection

Assessment points	1	2	3	4	5	6
Assessment point window	Cardiac arrest event	2 days (± 3 days) after assessment 1	Regular in-hospital reviews	30-day (± 2 days) after assessment 1	Hospital discharge†	Six-months (± 1 m) after assessment 1
Eligibility assessment	✓					
Intervention	✓					
Cardiac arrest event data	✓	✓				
Patient demographics/past medical history		✓				
Defibrillator record download		✓				
Post-cardiac arrest treatment		✓	✓	✓		
Length of hospital stay					✓	
Survival status	✓	✓	✓	✓	✓	✓
Quality of life (SF-12 and EQ-5D-5 L)					✓	✓
Assessment of blinding					✓	✓
Consent to continue‡		✓	✓	✓	✓	
Adverse events	✓	✓	✓	✓	✓	

† − May occur before visit four; ‡ − Seek at first appropriate opportunity

SF-12- 12-item short form survey; EQ-5D-5 L- EuroQOL- 5 dimensions- 5 levels

### Adverse events/adverse device events

COMPRESS-RCT enrols a population of participants who are in an immediately life-threatening situation. The majority of participants are not expected to survive, and those that do will require a prolongation of their hospital stay and may have long-term incapacity and disability. These events will be recorded as outcome measures. As such, adverse events and adverse device events will only be recorded where the event is potentially related to trial participation (i.e. may have resulted from study treatment) and the event is unexpected. Events are only recordable if they occur prior to the participant being discharged from hospital.

Expedited reporting is required for recordable adverse events and adverse device events that meet standardised criteria for seriousness, namely events that: result in death, are immediately life-threatening, require hospitalisation or prolongation of existing hospitalisation, result in persistent or significant disability or incapacity, result in congenital abnormality or birth defect, or result in an important medical condition.

### Sample size

The planned sample size is 330 participants. This number is based on two separate calculations.

Firstly, in relation to the primary outcome, we project a total eligible sample size of 550 participants at study sites over the study period. Based on a recruitment rate of 60%, a sample size of 330 randomised patients will allow us to estimate the 95% confidence interval of the recruitment rate with sufficient precision (i.e. 55.9 to 64.1%) to support progression to an effectiveness trial.

Secondly, we will use the approach of comparing groups using an 80% one-sided confidence interval, as described by Cocks and Torgerson, to establish if a clinically meaningful difference between groups in an effectiveness trial can be ruled out, thereby precluding progression to an effectiveness trial [20]. For an effectiveness trial, a sample size of 3554 patients would be required to detect a 3.5% absolute improvement in 30-day survival at a power of 90% and a significance level of 0.05. As such, 9% of 3554 (320) is required for our feasibility study, which has been slightly increased to account for patients lost to follow-up [20].

### Statistical analysis

Feasibility outcomes will be reported using descriptive statistics. Categorical data will be described as frequency and percentage. Continuous data will be assessed for normality, and described as mean and standard deviation or median and interquartile range as appropriate.

We will report and analyse patient and process outcomes as we would for an effectiveness trial. Analyses will be undertaken on an intention-to-treat basis. For dichotomous outcomes, we will describe differences between groups as risk ratio and 95% confidence interval. For continuous outcomes, we will report mean difference and 95% confidence interval. In addition, for the outcome of 30-day survival, we will compare groups using an 80% one-sided confidence interval, based on the approach described by Cocks and Torgerson [20]. We will report both unadjusted analyses, and analyses adjusted for key baseline characteristics.

### Qualitative study

The qualitative aspect of the study will be undertaken at centres participating in the clinical trial, and will explore staff member’s experiences of being involved in the trial, as well as potential barriers and facilitators to recruitment. Following written informed consent, a researcher will conduct a digitally audio recorded face-to-face semi-structured interview with the staff member about their experience of the COMPRESS-RCT study. Staff members will be purposively sampled to ensure a diverse sample based on factors such as clinical role, study site, and whether or not a patient was actually randomised to the study. Recruitment will continue recruitment until data saturation is achieved, which is anticipated to be approximately 30 participants [21, 22].

Interviews will be transcribed verbatim. Data will be analysed using a constant comparative method that is informed by a grounded theory approach [23]. We will review each interview on its completion and compare it to other interview data. This process will enable us to identify patterns and themes within the dataset, and may facilitate the development of a conceptual model to describe potential and actual barriers to recruitment, together with possible solutions.

### Trial oversight/monitoring

The trial is managed by a trial management group that meets on a monthly basis. The management group is comprised of clinical co-applicants, a methodologist, administrative staff, and patient research ambassadors. Independent oversight is provided through a trial oversight committee, comprised of an independent chair and trial management group members. The oversight committee meets on a six-monthly basis. The oversight committee chair, trial statistician, and an independent clinical expert review unblinded outcome data in a closed meeting following each oversight committee meeting.

## Discussion

The time-critical nature of cardiac arrest makes research on the effectiveness of cardiac arrest interventions both practically and ethically challenging [24]. Observational studies provide useful insights in to the effectiveness of interventions, but are prone to biases, such as selection bias and the recently described resuscitation time bias [24, 25]. In the out-of-hospital setting, observational studies of mech-CPR have produced inconsistent findings [26–28]. In contrast, findings of high-quality large randomised controlled trials have produced consistent results, showing no benefit in the routine use of mech-CPR compared with man-CPR [10]. Such data highlights the value of using high-quality randomised controlled trials, where available, as a basis for policy-making.

To date, cardiac arrest randomised controlled trials have tended to focus on the out-of-hospital setting. A systematic review of cardiac arrest randomised controlled trials published between 2002 and 2012 identified 61 trials, of which only three (5%) included in-hospital cardiac arrest patients [29]. Differences between out-of-hospital and in-hospital cardiac arrest in terms of patient characteristics and clinical response limit the direct generalisability of research findings between these settings [1, 30]. Furthermore, it likely means that experience and lessons learned from the successful delivery of out-of-hospital cardiac arrest research cannot necessarily be directly transferred to the in-hospital setting [31].

COMPRESS-RCT will provide important new information as to the feasibility of conducting a randomised controlled trial of mech-CPR in the hospital setting. More broadly, we will provide new insight in to the challenges of both delivering cardiac arrest research on a 24/7 basis in an acute hospital.

### Trial status

The current approved protocol is version 3.0, dated 29th January 2018. The full protocol is included in the electronic supplement. Trial recruitment is ongoing. Trial recruitment commenced February 2017, with a scheduled end date of February 2019.

## Additional file

Additional file 1: Formulae for deriving study feasibility outcomes. (PDF 288 kb)

## Abbreviations

CPR: cardiopulmonary resuscitation
EQ-5D-5 L: EuroQOL- 5 dimensions- 5 levels
Man-CPR: manual cardiopulmonary resuscitation
Mech-CPR: mechanical cardiopulmonary resuscitation
SF-12: 12-item short form survey
