# Supplementary material for: Mechanical versus manual chest compressions in the treatment of in-hospital cardiac arrest patients in a non-shockable rhythm: a randomised controlled feasibility trial (COMPRESS-RCT)
Source: Scand J Trauma Resusc Emerg Med. 2018 Aug 30;26:70. doi: 10.1186/s13049-018-0538-6 (PMC6117876; doi:10.1186/s13049-018-0538-6)
Supplement: Supplementary file 1 — Formulae for deriving study feasibility outcomes. (PDF 288 kb) [file 13049_2018_538_MOESM1_ESM.pdf]

Appendix two- formulae for feasibility outcomes

Formula one:

$$Proportion\ of\ eligible\ patients\ randomised\ during\ operational\ hours = \frac{Number\ of\ randomised\ patients}{Number\ of\ eligible\ patients\ during\ operational\ recruitment\ hours}$$

Formula two:

$$Proportion\ of\ eligible\ patients\ randomised = \frac{Number\ of\ randomised\ patients}{Number\ of\ eligible\ patients}$$

Formula three:

$$Proportion\ of\ patients\ randomised\ outside\ of\ working\ hours = \frac{Number\ of\ patients\ randomised\ outside\ of\ working\ hours}{Number\ of\ randomised\ patients}$$

Formula four

$$Proportion\ of\ patients\ or\ consultees\ agreeing\ to\ ongoing\ study\ participation = \frac{Number\ of\ patients\ where\ option\ one\ or\ two\ selected}{Number\ of\ patients\ approached\ for\ agreement}$$

Formula five (measured at discharge and six-months)

$$Proportion\ of\ patients\ adequately\ blinded\ (1) = \frac{Number\ of\ patients\ reporting\ \text{definitely know}\ whether\ or\ not\ treated\ with\ mechanical\ device}{Number\ of\ patients\ completing\ questionnaire}$$

Formula six (measured at discharge and six-months)

$$Proportion\ of\ patients\ adequately\ blinded\ (2) = \frac{Number\ of\ patients\ accurately\ predicting\ treatment\ arm}{Number\ of\ patients\ completing\ questionnaire}$$

Formula seven (measured at discharge and six-months)

$$Proportion\ of\ patients\ with\ complete\ follow-up\ data = \frac{Number\ of\ patients\ with\ complete\ follow-up\ data}{Number\ of\ alive\ randomised\ patients}$$

Formula eight

$$Proportion\ of\ patients\ with\ analysable\ chest\ compression\ quality\ data = \frac{Number\ of\ patients\ with\ analysable\ chest\ compression\ quality\ data}{Number\ of\ randomised\ patients}$$
